# Factors Associated with Maternal Near Miss among Women Admitted in West Arsi Zone Public Hospitals, Ethiopia: Unmatched Case-Control Study

**DOI:** 10.1155/2020/6029160

**Published:** 2020-07-02

**Authors:** Fikadu Nugusu Dessalegn, Feleke Hailemichael Astawesegn, Nana Chea Hankalo

**Affiliations:** ^1^Department of Public Health, College of Medicine and Health Sciences, Madda Walabu University, Bale Goba, Ethiopia; ^2^School of Public Health, College of Medicine and Health Sciences, Hawassa University, Hawassa, Ethiopia

## Abstract

**Background:**

Maternal near miss refers to a very ill pregnant or delivered woman who nearly died but survived a complication during pregnancy, childbirth, or within 42 days of termination of pregnancy. Maternal death; the most catastrophic end is frequently described as just “tip of the iceberg,” whereas maternal near-miss as the “base.” Therefore, this study aimed at assessing the factors associated with maternal near-miss among women admitted in public hospitals of West Arsi zone, Ethiopia.

**Methods:**

A facility-based unmatched case-control study was conducted from Mar 1 to Apr 30, 2019. Three hundred twenty-one (80 cases and 241 controls) study participants were involved in the study. Cases were recruited consecutively as they present, whereas controls were selected by systematic sampling method. Cases were women admitted to hospitals during pregnancy, delivery, or within 42 days of termination of pregnancy and fulfilled at least one of the maternal near-miss disease-specific criteria, while controls were women admitted and gave birth by normal vaginal delivery. The interviewer-administered structured questionnaire and data abstraction tool was used to collect data. Data were entered Epi data 3.1 and then transferred into SPSS 20 for analysis. Multivariable logistic regression was used, and the significance level was declared at *p* value ≤ 0.05.

**Results:**

The major maternal near-miss morbidities were severe obstetric hemorrhage (32.5%), pregnancy-induced hypertensive disorders (31.3%), and obstructed labor (26.3%), followed by 6.3% and 3.8% of severe anemia and pregnancy-induced sepsis, respectively. The odds of maternal near miss were statistically significantly associated with women's lack of formal education [AOR = 2.24, 95% CI: (1.17, 4.31)]. Not attending antenatal care [AOR = 3.71, 95% CI: (1.10, 12.76)], having prior history of cesarean section [AOR = 3.53, 95% CI: (1.49, 8.36)], any preexisting chronic medical disorder [AOR = 2.04, 95% CI: (1.11, 3.78)], and having experienced first delay [AOR = 5.74, 95% CI: (2.93, 11.2)].

**Conclusions:**

Maternal education, antenatal care, chronic medical disorders, previous cesarean section, and first delay of obstetric care-seeking were identified as factors associated with maternal near-miss morbidity. Therefore, this finding implies the need to get better with those factors, to preclude severe maternal complications and subsequent maternal mortality.

## 1. Introduction

Maternal death is the most catastrophic end that could happen to a pregnant woman. It is frequently described as just “tip of the iceberg” while maternal morbidity as the “base,” and for every woman who dies, many more will survive but often suffer from lifelong disabilities [1, 2]. World Health Organization (WHO) defines the maternal near-miss event as “a woman who nearly died but survived a complication that occurred during pregnancy, childbirth or within 42 days of termination of pregnancy” [3, 4]. Thus, maternal near miss is increasingly used as an indicator of the quality of obstetric care and clinical practice. The practical implementation of this concept should provide a significant contribution to reduce maternal deaths and improve maternal health [4–6].

Globally, 303,000 maternal deaths were occurred in 2015, with the highest-burden being in sub-Saharan African countries [7]. In other words, women in sub-Saharan Africa have 1 in 39 risk of dying in childbirth compared to 1 in 3,800 in industrialized countries in their lifetime [5, 8]. Maternal mortality continues to be of great public health importance because many more women experience life-threatening complications [9, 10], during pregnancy, delivery, and postpartum complications [11]. Despite all the efforts on maternal health care, maternal near-miss, disabilities, and deaths were exceptionally high in developing countries, including Ethiopia [12].

Ethiopia is one of sub-Saharan Africa countries with the highest maternal mortality rate. According to the Ethiopian Demographic and Health Survey (EDHS, 2016) report, MMR is 412 per 100,000 live births, and for every maternal death, 10% to 15% of the women develop disability from pregnancy and pregnancy-related complications [13]. Ethiopia is one of five countries that account for half of the maternal deaths globally [14]; about 20,000 women die each year from pregnancy and childbirth complications [15]. A study done at Ayder Referral Hospital in Tigray showed that 22.7% near misses and MMR of 427 per 100,000 live births [16]. Besides, a retrospective review done in Ethiopia at Debre-Markos referral hospital found that 403 (29.7%) near-miss cases from a total of 1355 case notes reviewed in a five-year period [17].

Maternal near-miss complications are numerous and are estimated to be around 12 times more frequent than maternal deaths in Ethiopia [18]. However, the factors associated with it are not well-studied using the standardized WHO criteria to measure maternal near-miss. In addition, previously published studies conducted in the country relied on patient records review to assess factors of maternal near-miss [15–18]. Hence, these studies might be subjected to information bias due to incompleteness and poor quality of secondary data at the health facility. And also, the study design used was cross-sectional that has known limitations of ascertaining cause-effect relationships [16, 18]. Therefore, this study was carried out to assess factors associated with maternal near-miss among women admitted in public hospitals of West Arsi zone, Oromia region, Ethiopia, in 2019. The evidence generated through this study would be used by the local health planner, stakeholders working on maternal health programs.

## 2. Materials and Methods

### 2.1. Study Setting and Period

The study was conducted in West Arsi zone public hospitals from Mar 1 to Apr 30, 2019. The zones are found in Oromia regional state, Ethiopia. According to the 2007 national household census, the zone has a total population of 1,964,038, of whom 990,295 are women. The total number of women of reproductive age (15-49 years) is estimated to be 434,412 [19]. In the zone, there are four government hospitals, Shashemene Referral Hospital, Melka Oda General Hospital, Dodola General Hospital, and Kokosa Primary Hospital. And three private/NGO hospitals (Negele Arsi General Hospital, Feya Primary Hospital, and Gambo Primary Hospital). Additionally, there are 81 functional health centers, 351 functional health posts, 179 private clinics, 1NGO clinic, and 95 pharmacy/drug shop.

### 2.2. Study Design

An unmatched case-control study design was employed.

### 2.3. Source and Study Populations

Women who were admitted at selected hospitals during pregnancy, labor, or within the first 42 days of termination of pregnancy.

#### 2.3.1. Selection of Cases

Cases were women who admitted to the hospitals due to pregnancy-related complications, deliver/abortion, or within 42 days of termination of pregnancy. And those who fulfill at least one of the five diagnostic maternal near-miss validated disease-specific criteria proposed by WHO, Filippi et al., and Say et al. [4, 20, 21]. Obstructed labor (uterine rupture, impending rupture like prolonged labor with previous C.S., emergency C.S.), hemorrhage (severe obstetric hemorrhage leading to shock, emergency hysterectomy, coagulation defects, and/or blood transfusion of at least one units), pregnancy-induced hypertension disorders (severe preeclampsia or eclampsia), sepsis (septic abortion, infections including hyper or hypothermia or a clear source of infection and clinical signs of septic shock), and severe anemia (including low hemoglobin *<*6 g/dl or clinical signs of severe anemia in women without hemorrhage).

#### 2.3.2. Selection of Controls

Controls were those women who admitted to the same hospital with normal labor and gave birth in normal vaginal delivery without complications.

### 2.4. Sample Size Determination

The sample size was calculated using Epi Info version 7 software package designed for an unmatched case-control study. The following assumptions were made during calculating sample size: 95% confidence level, 80% power, and a case to control the ratio of 1 : 3. The sample size calculated for main exposure variables associated with near maternal miss using variables from different kinds of literature. Then, variable resulted in a high sample size were taken. Delay to reach the place of health care was the main exposure variable for maternal near miss that provided the maximum sample size, which was taken from the study done in Northern Ethiopia [22]. According to the study, 40.5% of controls delay for >60 minutes in reaching the final place of care, while 60.2% of cases delay for >60 minutes. This gives a total sample size of 292 (73 cases and 219 controls). By taking a 10% nonresponse rate, the final sample size was 321 (80 cases and 241controls).

### 2.5. Sampling Technique

The sample size was proportionally allocated for each public hospitals in the zone based on their number client flow. Then, cases were recruited consecutively as they present, whereas controls were selected by systematic sampling method with an interval of five (*k* = 1309/241 = 5).

### 2.6. Data Collection Tools

The questionnaire and near-miss data abstraction tools were adapted from different literature [4, 15, 21]. For the identification of cases, the WHO disease-specific criteria were used. The near-miss data abstraction tool was constructed for the case and control identification from medical records. The questionnaire was composed of four main parts: -mothers' socioeconomic and demographic factors, reproductive health and obstetric characteristics, previous chronic medical history, and obstetric health care delays.

### 2.7. Data Collection Technique and Procedure

Exit interviews were conducted by trained data collectors using structured; pretested questionnaires. Identifying cases and collecting information which could not be obtained by interview like the diagnosis of obstetric complications, laboratory investigation, and management were extracted from patient medical records and discharge summaries. Eight midwife nurses who have experience on obstetric care (two midwives per hospital) and who can speak the local language were recruited as data collectors. Four nurses who have bachelor's degree holders were recruited as supervisors.

### 2.8. Operational Definitions

#### 2.8.1. Maternal Near Miss

Admitted women with at least one of the following obstetric diagnosis: severe preeclampsia or eclampsia, severe hemorrhage, dystocia (uterine rupture, impending uterine rupture like prolonged labor with previous cesarean section, and emergency C.S. delivery), severe anemia (hemoglobin<6 g/dl), and sepsis (puerperal sepsis, chorioamnionitis, and septic abortion) [3, 4, 21].

#### 2.8.2. The First Phase (Referral Status)

The period between arrival at the first health facility and arrival at the current study hospital. Accepted time spent between arrival and the first examination is usually set not more than 60 minutes.

#### 2.8.3. The Second Phase

The time spent between arrival at the final current study hospital and the first examination, followed by the time spent between the first examinations and receiving the first care. Accepted time spent between examination and receiving first care is usually set not more than 30 minutes. Therefore, the third delay is a delay in at least one phase delay of the two-third delay phases. The delay in referral from various health facilities and multiple referrals were included in the third delay within intermediate health facilities.

#### 2.8.4. Well-Birth Prepared

Defined as having taken at least 3 of the four actions (bought childbirth materials, saved money, identified transport, identified skilled provider or health facility).

### 2.9. Data Quality Assurance Techniques

Training was given for data collectors and supervisors for one day on interview techniques, confidentiality of the information, and informed consent. The pretest was conducted in Robe hospital, using 5% of the sample size who fulfill the inclusion criteria, and feedbacks were incorporated accordingly. The data completeness and consistency were checked by supervisors on a daily basis.

### 2.10. Data Management and Analysis

The data were entered and cleaned using Epi data version 3.1, then exported to SPSS version 20 for further analysis. Univariate analysis: proportions, frequencies, and averages were calculated for study variables to compare cases and controls. Bivariable and multivariable logistic regression were to identify predictor variables for maternal near miss. All variables having a *p* value ≤ 0.25 in the bivariate analysis were considered for multivariable logistic regression model. The Hosmer and Lemeshow goodness of fit test was used to determine whether the model adequately describes the data and the model adequately fitted for the final model (*p* = 0.108). Confounders were controlled with multivariable logistic regression, and multicollinearity was also checked with a correlation matrix. Adjusted odds ratio (AOR) with 95% CI was estimated to assess the presence and strength of associations, and statistical significance was declared at a *p* value ≤ 0.05.

## 3. Results

### 3.1. Sociodemographic and Economic Characteristics of Women

The mean (±SD) age of cases and controls was 27.56 (±6.0) and 27.0 (±5.43) years, respectively. However, the mean age difference between cases and controls was not statistically significant *p* = 0.105. The majority of cases were an urban residence, 53 (66.2%), and nearly half of controls 116 (48.1%) were rural residents. Of the total respondents, 294 (91.6%) of them were currently in marital union. Concerning educational status, 38 (47.5%) cases and 51 (21.2%) controls had no formal education. Regarding transportation, 48 (60%) cases and 180 (74.7%) controls have access to transportation (Table 1).

### 3.2. Reproductive and Obstetric Related Characteristics of Study Participants

The proportion of early marriage among near-miss groups and control groups was comparable 31.2% and 29.2%, respectively. In terms of the history of caesarian section, 18 (22.5%) cases and 21 (8.7%) controls had at least one history of the previous caesarian section. Likewise, the history of prior abortion was 6.2% and 12% among cases and controls, respectively (Table 2).

### 3.3. Preexisting Chronic Medical Disorders and Maternal Obstetric Health Care Delays

History of at least one chronic preexisting medical disorder was reported in half (51.2%) of cases and one third (32.8%) of controls. Majority 220 (68.5%) were self-referred from home, while 40% of cases and 28.6% of controls referred from health facilities, respectively.

More than half (55%) of near-miss cases delayed >4 hours for deciding to go to the health facility compared to controls (12.4%) (Table 3).

### 3.4. Clinical Characteristics of Near Misses

**Figure 1:**
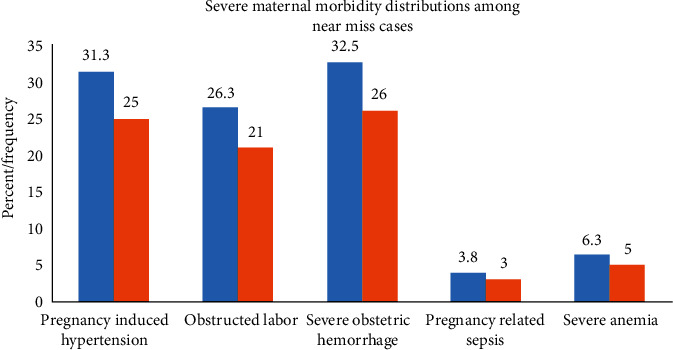
Maternal morbidity among near-miss cases admitted in Western Arsi zone public hospitals, Ethiopia, 2020.

Among 80 near-miss cases, the most complications were severe obstetric hemorrhage(32.5%), followed by severe pregnancy-induced hypertensive disorders (31.3%). Most of near misses (79%) had occurred before hospital admission. The remaining 21% occurred during or after admission to the hospitals (Figure 1).

### 3.5. Factors Associated with Maternal Near Misses among Women Admitted in Public Hospitals

Women with no formal education had two times [AOR = 2.24, 95% CI: (1.17, 4.31)] higher odds of developing maternal near-miss compared to women with formal education. Similarly, women who did not have an antenatal care follow-up had 3.71 times [AOR = 3.71, 95% CI: (1.10, 12.76)] greater odds of developing maternal near-miss compared to women who had four and more antenatal visits. The odds of maternal near-miss was 3.53 times [AOR = 3.53, 95% CI: (1.49, 8.36)] greater among women with a history of previous cesarean section as compared to women who had no history of previous cesarean section.

Moreover, women who had at least one preexisting medical disorder had two times [AOR = 2.04, 95% CI: (1.11, 3.78)] increased odds of maternal near miss compared to their counterparts. Delay to seek obstetric health care (first delay) was strongly associated with maternal near miss. The odds of maternal near-miss among women who delayed more than 4 hours at home were 5.74 times higher compared to those who decided to seek health care within 4 hours [AOR = 5.74, 95% CI: (2.93, 11.2)] (Table 4).

## 4. Discussion

In this study, women who did not have formal education had higher odds of near maternal miss compared to those who had formal education. This is comparable with studies in Bolivia, Morocco, Brazil, and Northern Ethiopia [19, 22–24], where illiterate mothers had higher odds of the maternal near miss. Possibly women with no formal education lack access to relevant information, which in one or another way may influence mothers' awareness of the obstetric complications and the need to seek better medical services. However, studies were done in Uganda, Ile-Ife Nigeria, Northeast Brazil, and Erbil city. Iraq [25–28] showed no significant association between women's education and maternal near-miss, and this is might be due to the study setting and study time difference.

Lack of antenatal care visits was the strong Factor associated with maternal near-miss in this study. This finding was parallel with the studies conducted in Bolivia, Pakistan, Morocco, and Iraq [19, 23, 25, 29, 30]. The study in Nigeria and Jimma University teaching hospital in Ethiopia also confirm the optimal number of antenatal care attendance as a protective factor against the severe maternal outcome and near miss. Furthermore, studies in Northeast Brazil as well as at Ayder teaching hospital Mekelle and Debra-Markos referral hospital in Ethiopia showed that women who had no antenatal visits were more likely to develop maternal near-miss [16, 17, 28]. This might be antenatal care is the most favorable contact point for mothers to get more information about the pregnancy. As well as discussion with health professionals on danger signs of pregnancy and delivery.

Consistence with previous studies in Netherland, Northeast Brazil South Africa, and Ethiopia [20, 28, 31–33], there was a higher risk of maternal near-miss among women with a prior history of cesarean section compared to their counterparts. Nevertheless, a study done in Tanzania showed that previous cesarean deliveries and maternal near-miss has no association [34]. This might be due to the difference in population, number, and quality of the recurrent cesarean section, and health service delivery systems. Giving the impression for a previous caesarian section delivery is critical because this kind of delivery, most of the time justified in case of health threats, which perhaps reoccur during the mother's subsequent pregnancy. As well, the previous cesarean section leads to increased risks of uterine rupture and hemorrhage due to uterine scar tissue during the next vaginal delivery. This implies cesarean section delivery especially elective nonmedical cesarean section should have to be reduced to the acceptable level [35] and avoid the misconception “once a cesarean section, always a cesarean section” to reduce the threats related with delivery.

Maternal near-miss was significantly associated with a history preexisting chronic medical disorder. This finding is comparable with studies in Iraq, Netherlands, and Uganda [25, 27, 32]. It was reported that the history of anemic resulted in maternal near maternal miss in countries like Ghana, Nigeria, and Sudan [26, 36–38]. Similarly, the history of prior chronic hypertension was associated with a high risk of maternal miss from studies in Brazil, Nigeria, and Ethiopia [26, 39]. In contrast, a study carried out on “applying the new concept of a maternal near-miss in an intensive care unit” illustrates preexisting medical morbidity has not increased the risk of maternal near-miss [40]. This inconsistency might be due to the difference in the approach used to diagnose near-miss cases like exclusively using management-based diagnosis (admission to intensive care unit) in such studies.

Likewise, the present study revealed that near-miss cases women were more likely to have a first delay (delay in deciding for health care) than their counterparts. This is in line with studies done in Morocco, Ile-Ife Nigeria, and Ethiopia [19, 26, 31]. Whereas second and third delays were not significantly associated with maternal near miss. This might be the fact that second and third delays would be more or less managed with improved ambulance services and due to the availability of enhanced comprehensive obstetric services in the current study hospitals as per the Ethiopian Ministry of Health recommendation. Finally, the findings of this study should be interpreted in light of its limitation. Although a disease-specific criterion was used to ascertain near-miss cases as per WHO recommendation for developing countries, the identification of cases using such criteria is less rigorous. In addition, there might be misclassification bias even though the identified cases were verified by senior physicians working in the study hospitals.

## 5. Conclusion

This study identified that women's with no formal education, lack of antenatal care visits, preexisting maternal chronic medical disorders, history of previous cesarean section, and first delay of obstetric care-seeking were factors associated with a maternal near miss. These findings had better recommend in rural areas, especially where there are high numbers of mothers with no formal education; focusing on the coverage of maternity service is a crucial step to avert serious maternal complications through strengthening health extension packages and scaling up of antenatal care. Furthermore, health facilities found in the zone better provide a quality antenatal care in order to identify high-risk women and prevent maternal near-miss morbidity. Those women who had a prior history of cesarean section and any preexisting medical disease must actively be recognized during pregnancy to prevent occurrence of maternal near miss. Finally, we recommend a longitudinal multicentre study to generate a more stable and more comprehensive national illustration of the maternal near miss.

## Figures and Tables

**Table 1:** Sociodemographic and economic characteristics of women admitted in Western Arsi zone public hospitals, Ethiopia, 2020.

Characteristics	Cases (*n* = 80)	Controls (*n* = 241)	Total (*n* = 321)	Chi-square	*p* value
Residence					
Rural	53 (66.2)	116 (48.1)	169 (52.6)	7.908	0.005
Urban	27 (33.8)	125 (51.9)	152 (47.4)		
Women's age					
<20 year	9 (11.2)	29 (12.0)	38 (11.8)	4.499	0.105
20-34 year	52 (65.0)	179 (74.3)	231 (72.0)		
≥35 year	19 (23.8)	33 (13.7)	52 (16.2)		
Mean (±SD)	27.56 (±6.0)	27.0 (±5.43)			
Current marital status					
Not in marital union	8 (10.0)	19 (7.9)	27 (8.4)	0.349	0.555
In marital union	72 (90.0)	222 (92.1)	294 (91.6)		
Women's education					
No formal education	38 (47.5)	51 (21.2)	89 (27.7)	24.97	<0.001
Primary education	29 (36.2)	98 (40.7)	127 (39.6)		
Secondary education	11 (13.8)	63 (26.1)	74 (23.0)		
Higher education	2 (2.5)	29 (12.0)	31 (9.7)		
Husband's education (*n* = 313)					
No formal education	21 (27.3)	55 (23.3)	76 (24.3)	5.299	0.151
Primary education	32 (41.5)	78 (33.0)	110 (35.1)		
Secondary education	16 (20.8)	53 (22.5)	69 (22.0)		
Higher education	8 (10.4)	50 (21.2)	58 (18.6)		
Monthly income					
≤1000 ETB	29 (36.2)	57 (23.7)	86 (26.8)	5.639	0.131
1001–2000 ETB	15 (18.8)	46 (19.0)	61 (19.0)		
2001–3000 ETB	12 (15.0)	38 (15.8)	50 (15.6)		
≥3001 ETB	24 (30.0)	100(41.5)	124 (38.6)		
Distance to nearest F.H.					
>60 minutes	31 (38.8)	51 (21.2)	82 (25.5)	9.769	0.002
≤60 minutes	49 (61.2)	190 (78.8)	239 (74.5)		
Road access					
Yes	61 (76.2)	201 (83.4)	262 (81.6)	2.048	0.152
No	19 (23.8)	40 (16.6)	59 (18.4)		
Transportation access					
Yes	48 (60.0)	180 (74.7)	228 (71.1)	5.305	0.021
No	32 (40.0)	61 (25.3)	93 (28.9)		

**Table 2:** Reproductive and obstetric characteristics of women admitted in Western Arsi zone public hospitals, Ethiopia, 2020.

Characteristics	Cases (*n* = 80)	Controls (*n* = 241)	Total (*n* = 321)	Chi-square	*p* value
ANC visits					
No visit at all	47 (58.8)	48 (19.9)	95 (29.6)	43.469	<0.001
At least one visit	33 (41.2)	193 (80.1)	226 (70.4)		
Timing of ANC booking (*n* = 226)					
Early booking (≤12 weeks)	6 (18.2)	57 (29.5)	63 (27.9)	1.806	0.179
Late booking (>12 weeks)	27 (81.8)	136 (70.5)	163 (72.1)		
Gravidity					
1	27 (33.8)	44 (18.3)	71 (22.1)	8.369	0.015
2-4	33 (41.2)	123 (51.0)	156 (48.6)		
≥5	20 (25.0)	74 (30.7)	94 (29.3)		
Parity					
0	27 (33.8)	52 (21.6)	79 (24.6)	4.957	0.084
1-4	36 (45.0)	134 (55.6)	170 (53.0)		
≥5	17 (21.2)	55 (22.8)	72 (22.4)		
Ever had abortion					
Yes	5 (6.2)	29 (12.0)	34 (10.6)	2.121	0.145
No	75 (93.8)	212 (88.0)	287 (89.4)		
History of previous cesarean section					
Yes	18 (22.5)	21 (8.7)	39 (12.1)	10.695	0.001
No	62 (77.5)	220 (91.3)	282 (87.9)		
Undergone female genital mutilation					
Yes	60 (75.0)	170 (70.5)	230 (71.7)	0.588	0.443
No	20 (25.0)	71 (29.5)	91 (28.3)		
Age at first marriage(*n* = 313)					
Age ≤ 18 years	24 (31.2)	69 (29.2)	93 (29.7)	0.104	0.747
Age > 18 years	53 (68.8)	167 (70.8)	220 (70.3)		
Age at first pregnancy					
<16	9 (11.2)	12 (5.0)	21 (6.5)	3.887	0.143
16-19	33 (41.2)	104 (43.2)	137 (42.7)		
≥20	38 (47.5)	125 (51.9)	163 (50.8)		
Birth interval (*n* = 268)					
≥24 months	44 (83.0)	176 (81.9)	220 (82.1)	0.039	0.844
<24 months	9 (17.0)	39(18.1)	48 (17.9)		
Current pregnancy planned					
Yes	66 (82.5)	209 (86.7)	275 (85.7)	0.872	0.350
No	14 (17.5)	32 (13.3)	46 (14.3)		
Well birth prepared					
Yes	39 (48.8)	123 (51.0)	162 (50.5)	0.126	0.723
No	41 (51.2)	118 (49.0)	159 (49.5)		

**Table 3:** Preexisting chronic medical disorders and maternal obstetric health care delays among women admitted in Western Arsi zone public hospitals, Ethiopia, 2020.

Characteristics	Cases (*n* = 80)	Controls (*n* = 241)	Total (*n* = 321)	Chi-square	*p* value
Referral status					
Health facility referred	32 (40.0)	69 (28.6)	101 (31.5)	3.600	0.058
Self-referred from home	48 (60.0)	172 (71.4)	220 (68.5)		
Means of transportation					
Ambulance	32 (40.0)	73 (30.3)	105 (32.7)	2.573	0.109
Other than ambulance^∗∗^	48 (60.0)	168 (69.7)	216 (67.3)		
First delay					
Yes	44 (55.0)	30 (12.4)	74 (23.1)	61.308	<0.001
No	36 (45.0)	211 (87.6)	247 (76.9)		
Second delay					
Yes	24 (30.0)	56 (23.2)	80 (24.9)	1.468	0.226
No	56 (70.0)	185 (76.8)	241 (75.1)		
Third delay					
Yes	21 (26.2)	54 (22.4)	75 (23.4)	0.495	0.481
No	59 (73.8)	187 (77.6)	246 (76.6)		
Previous chronic hypertension					
Yes	16 (20.0)	26 (10.8)	42 (13.1)	4.482	0.034
No	64 (80.0)	215 (89.2)	279 (86.9)		
Previous anemia					
Yes	22 (27.5)	40 (16.6)	62 (19.3)	4.581	0.032
No	58 (72.5)	201(83.4)	259 (80.7)		
HIV positive					
Yes	9 (11.2)	12 (5.0)	21 (6.5)	3.863	0.049
No	71 (88.8)	229 (95.0)	300 (93.5)		
History of maternal cardiac disease					
Yes	12 (15.0)	15 (6.2)	27 (8.4)	6.005	0.014
No	68 (85.0)	226 (93.8)	294 (91.6)		
History of diabetic mellitus					
Yes	9 (11.2)	17 (7.1)	26 (8.1)	1.421	0.233
No	71 (88.8)	224 (92.9)	295 (91.9)		
At least one preexisting medical problem					
Yes	41 (51.2)	79 (32.8)	120 (37.4)	8.753	0.003
No	39 (48.8)	162 (67.2)	201 (62.6)		

^∗∗^Other than ambulance includes public transport, private transport, on the walk, and carried by men.

**Table 4:** Factors associated with maternal near miss in multivariable logistic regression analysis, among women in West Arsi zone public hospitals, Ethiopia, 2019.

Factor variables	Maternal near miss		COR (95% CI)	AOR (95% CI)
	Cases (*n* = 80)	Control (*n* = 241)		
Place of residence				
Rural	53 (66.2)	116 (48.1)	2.11 (1.24, 3.58)	0.64 (0.31, 1.34)
Urban®	27 (33.8)	125 (51.9)	1.00	1.00
Maternal education				
No formal education	38 (47.5)	51 (21.2)	3.37 (1.97, 5.76)	2.24 (1.17, 4.31) ∗∗
Formal education®	42 (52.5)	190 (78.8)	1.00	1.00
Monthly income				
≤1000 ETB	29 (36.2)	57 (23.7)	2.12 (1.13, 3.98)	1.81 (0.79, 4.08)
1001–2000 ETB	15 (18.8)	46 (19.0)	1.36 (0.65, 2.82)	1.61 (0.64, 4.07)
2000–3000 ETB	12 (15.0)	38 (15.8)	1.32 (0.59, 2.89)	2.22 (0.84, 5.87)
≥3001® ETB	24 (30.0)	100 (41.5)	1.00	1.00
Distance to nearest facility				
>60 minutes	31 (38.8)	51 (21.2)	2.35 (1.36, 4.07)	1.14 (0.46, 2.78)
≤60 minutes®	49 (61.2)	190 (78.8)	1.00	1.00
Transportation access				
No	32 (40.0)	61 (25.3)	*1.96 (1.15, 3.35)*	0.78 (0.35, 1.74)
Yes®	48 (60.0)	180 (74.7)	1.00	1.00
ANC visit				
0	47 (58.8)	48 (19.9)	10.3 (3.41, 30.9)	3.71 (1.1, 12.76)^∗∗^
1	11 (13.7)	36 (14.9)	3.21(0.94, 10.92)	2.48 (0.66, 9.37)
2-3	18 (22.5)	115 (47.8)	1.64 (0.53, 5.13)	0.92 (0.26, 3.27)
≥4®	4 (5.0)	42 (17.4)	1.00	1.00
History of previous C.S.				
Yes	18 (22.5)	21 (8.7)	3.04 (1.53, 6.10)	3.53 (1.49, 8.36)^∗∗^
No®	62 (77.5)	220 (91.3)	1.00	1.00
Preexisting medical disorders				
Yes	41 (51.2)	79 (32.8)	2.16 (1.29, 3.61)	2.04 (1.11, 3.78)^∗∗^
No®	39 (48.8)	162 (67.2)	1.00	1.00
Means of transportation				
Ambulance	32 (40.0)	73 (30.3)	1.53 (0.91, 2.59)	1.76 (0.93, 3.31)
Not ambulance®	48 (60.0)	168 (69.7)	1.00	1.00
Referral status				
Health facility referred	32 (40.0)	69 (28.6)	1.66 (0.98, 2.81)	1.47 (0.72, 3.01)
Self-referred from home®	48 (60.0)	172 (71.4)	1.00	1.00
First delay				
Yes	44 (55.0)	30 (12.4)	8.59 (4.79, 15.4)	5.74 (2.93, 11.2)^∗∗^
No®	36 (45.0)	211 (87.6)	1.00	1.00

^∗∗^Statistically significant variables in multiple logistic regressions at *p* value ≤ 0.05. ®: Reference category.

## Data Availability

Data is not available for online access. However, readers who wish to gain access to the data can write to the corresponding author Feleke Hailemichael Astawesegn at felekeh86@gmail.com.

## Abbreviations

ANC: Antenatal care
AOR: Adjusted odds ratio
COR: Crude odd ratio
C.S.: Cesarean section
EDHS: Ethiopia demographic and health survey
MMR: Maternal mortality ratio
MNM: Maternal near miss
SDG: Sustainable development goal
WHO: World Health Organization.
